# Tooth Formation: Are the Hardest Tissues of Human Body Hard to Regenerate?

**DOI:** 10.3390/ijms21114031

**Published:** 2020-06-04

**Authors:** Juliana Baranova, Dominik Büchner, Werner Götz, Margit Schulze, Edda Tobiasch

**Affiliations:** 1 Department of Biochemistry, Institute of Chemistry, University of São Paulo, Avenida Professor Lineu Prestes 748, Vila Universitária, São Paulo 05508-000, Brazil; jbaranova@usp.br; 2 Department of Natural Sciences, Bonn-Rhein-Sieg University of Applied Sciences, von-Liebig-Straße 20, 53359 Rheinbach, NRW, Germany; dominik.buechner@h-brs.de (D.B.); margit.schulze@h-brs.de (M.S.); 3 Oral Biology Laboratory, Department of Orthodontics, Dental Hospital of the University of Bonn, Welschnonnenstraße 17, 53111 Bonn, NRW, Germany; wgoetz@uni-bonn.de

**Keywords:** dentogenesis, amelogenesis, dentinogenesis, cementogenesis, drug release materials, scaffolds, odontogenic cells, stem cells, whole-tooth regeneration

## Abstract

With increasing life expectancy, demands for dental tissue and whole-tooth regeneration are becoming more significant. Despite great progress in medicine, including regenerative therapies, the complex structure of dental tissues introduces several challenges to the field of regenerative dentistry. Interdisciplinary efforts from cellular biologists, material scientists, and clinical odontologists are being made to establish strategies and find the solutions for dental tissue regeneration and/or whole-tooth regeneration. In recent years, many significant discoveries were done regarding signaling pathways and factors shaping calcified tissue genesis, including those of tooth. Novel biocompatible scaffolds and polymer-based drug release systems are under development and may soon result in clinically applicable biomaterials with the potential to modulate signaling cascades involved in dental tissue genesis and regeneration. Approaches for whole-tooth regeneration utilizing adult stem cells, induced pluripotent stem cells, or tooth germ cells transplantation are emerging as promising alternatives to overcome existing in vitro tissue generation hurdles. In this interdisciplinary review, most recent advances in cellular signaling guiding dental tissue genesis, novel functionalized scaffolds and drug release material, various odontogenic cell sources, and methods for tooth regeneration are discussed thus providing a multi-faceted, up-to-date, and illustrative overview on the tooth regeneration matter, alongside hints for future directions in the challenging field of regenerative dentistry.

## 1. Introduction

Dental injuries and diseases such as caries and periodontitis are affecting significant fractions of populations worldwide and are the main reason for dental tissue regeneration efforts [1,2]. Caries lesions cause local enamel resorption and dentin damage due to oral microbiota activities in the morbid tooth. Although relatively easily manageable at early stages, if left untreated caries causes excessive dentin damage and poses a need for reparative treatment [3]. Periodontitis is a complex inflammatory disease, where pathogenic oral microbiota and host immune response dysregulation lead to the gingiva, periodontal ligament, cementum, and alveolar bone damage [4]. Excessive periodontitis damage cannot be regenerated naturally, thus requires specialized soft and hard calcified tissues regeneration approaches. Next to infectious/inflammatory oral diseases, several heritable disorders of dental tissue formation exist (e.g., amelogenesis imperfecta, dentinogenesis imperfecta, and tooth agenesis), which affect tooth formation, eruption, calcification, or maturation [5,6,7,8]. In addition to disrupted teeth integrity, dental diseases often create an unaesthetically looking oral cavity, thus affecting patients emotionally, which makes dental tissues regeneration critical in both aspects: health and aesthetics.

Dental tissues have no or very limited capacity for self-regeneration [2,3,9,10]. Specifically, enamel becomes acellular after it is formed; dentin regeneration is limited and dependent on the dental pulp stem cell pool, which deteriorates in the case of an infection and inflammation; and cementum has no remodeling capacity and limited regrowth in the case of disease-induced resorption [10,11,12,13]. Each dental tissue contains a defined amount of inorganic matter (hydroxyapatite crystals), matrix proteins arranged in a scaffolding network, and microstructures such as lacunae in cellular cementum and microchannels, which accommodate cellular processes in dentin and cementum. The complex microarchitecture of the tooth poses a need for appropriate replacement materials, which have to be biocompatible and wear-resistant [14]. Additionally, the development of enamel and dentin relies heavily on mesenchymal–epithelial interactions, thus making it challenging to recapitulate the process in vitro even using existing odontogenic cell lines and adult stem cell culture methods [10,12,15,16,17]. Although a lot is already known about tooth formation and molecular cues shaping this process [5,6,18], signaling patterns involved in dental tissue differentiation in vitro, postnatal calcified tissue metabolism and regeneration are being actively studied and more research is expected in the future [18,19,20,21,22,23,24,25,26,27,28,29,30,31,32,33,34,35,36,37,38,39,40,41,42,43,44,45,46,47].

Efforts in whole tooth regeneration have been made for decades [48] and include biological, bioengineering, and genetic approaches. Revitalizing the odontogenic potency of the successional dental lamina (SDL) rudiment for lost tooth regeneration might be one possibility to induce tooth formation in vivo in the adult [49]. Whole-tooth restoration using autologous tooth germ cells and bioengineered tooth germ transplantation is another promising opportunity [50,51]. However, due to limited sources of tooth germ cells, the risk for immune rejection of allogeneic or xenogeneic cells, as well as ethical and legal constraints, adult stem cells of various sources or induced pluripotent stem cells (iPSCs) may be used instead [52,53,54]. Recently, combining cells of mesenchymal and epithelial origin of various plasticity is being actively explored for tooth regeneration using novel culture methods [55,56,57]. 

Although the implantation of recombined embryonic or adult cells may give rise to tooth-like organs in vivo, the combination with scaffold material may improve tooth formation. Scaffolds can influence the biological behavior of cells and can give mechanical support to tissue constructs. Their consecutive degradation should parallel the formation of the native extracellular matrix and promote the assimilation of constructs after implantation [58]. In contrast to periodontal bone and other bone grafts, where numerous scaffold compounds have been developed and tested within the last decade [59,60,61,62,63,64], studies on artificial scaffolds for tooth regeneration are still rare due to the rather complex nature of teeth [14,65]. Recent studies in biomaterial development involve hybrids and composites of inorganic/organic components to be used as scaffolds to mimic the complex composition of the natural tooth [55,58,66]. New investigations have shown also that the functionalization of scaffolds using cell-free methods is possible. Vesicles, small RNAs, or exosomes from cultured stem cells or embryonic cells can be used onto or within scaffold material to address regenerative functions [65]. Besides, scaffolds can be loaded with drugs, growth factors, and/or receptor ligands to guide the stem cell differentiation process during dentogenesis [19,64,67,68]. However, very few artificial materials have been tested thus far in clinical trials [9,69].

In this review, the most recent discoveries regarding cellular signals guiding dental tissue differentiation in vitro and in vivo are summarized. Current developments of biocompatible functionalized scaffolds, drug-release materials, and their applications are addressed as well. Finally, whole-tooth generation approaches using various cellular sources and dilemmas in tooth regeneration are elucidated. An interdisciplinary approach is taken to cover tooth regeneration issues from molecular, via structural to biological aspects.

## 2. Hard Dental Tissues and Their Genesis

### 2.1. The Complexity of Dental Tissues

The process of teeth formation starts within embryogenesis and proceeds in multiple phases throughout the prenatal period, childhood and adolescence resulting in an eruption of permanent teeth. Each dental tissue forms in a unique way and in a tightly regulated manner, where one tissue is guiding or supporting the formation of the other [11,30,40]. Early odontogenesis is characterized by an epithelial–mesenchymal interaction, which is also a blueprint for the formation of other organs such as hair follicles or exocrine glands [70]. The epithelium is derived from the embryonic endoderm, while the mesenchyme is derived from the cranial neural crest. Placodal thickenings of the oral epithelium along the dental lamina first induce a cellular condensation of the underlying mesenchyme. The tooth primordium then undergoes different morphological stages forming a bud, cap, and later bell stage. While the epithelium gives rise to enamel, the mesenchyme is the source of the later pulp, periodontal apparatus and hard substances such as dentine and cementum. Then, epithelial components lose their inductive odontogenic competence while a reciprocal induction starts from the mesenchyme. These reciprocal crosstalks are governed by a signaling program consisting of a large number of molecules interacting in signaling pathways. Major examples of these factors are families such as the Bone Morphogenic Proteins (BMPs), Fibroblast Growth Factors (FGFs), Wingless/Int1 (Wnt), Hedgehog (Hh), or Ectodysplasin (EDA) functioning as morphogenetic inducers [18,65,71]. The morphogenesis is driven by signaling centers, which orchestrate tissue interactions and are involved in the size and shaping of the single tooth. In addition to cellular signaling, tissue forces, e.g., through an epithelial contraction, mesenchymal condensation, or bone biomechanics, participate in the formation of tooth morphology [71,72].

During tooth development, several stem cell niches have been identified. Epithelial stem cells are located, e.g., in the cervical loop, which is the apical end of the advancing epithelium consisting of an outer and inner layer and active until the onset of tooth root formation. These stem cells play a role in continuously growing teeth, e.g., mouse incisors. The elongation of the cervical loop as a double-layered structure is named Hertwig’s epithelial root sheath and is the signaling center for tooth root formation. It should also be mentioned that tooth formation depends on the interaction with the developing alveolar bone, which therefore should be considered in strategies for whole tooth regeneration [71,73]. 

**Figure 1:**
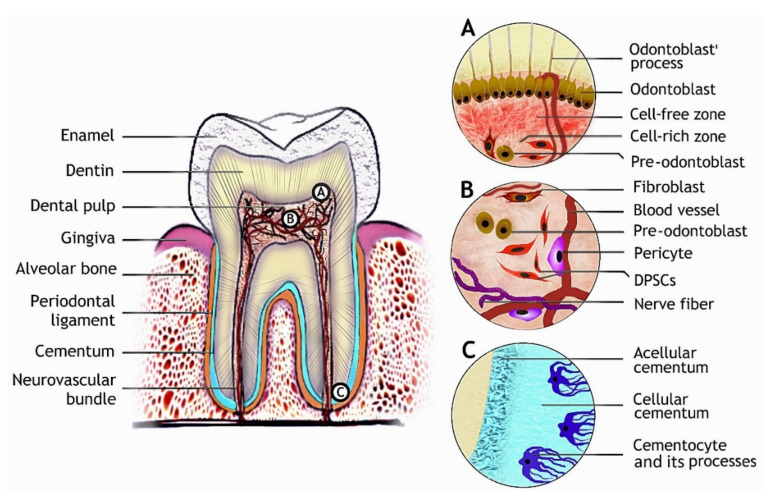
Tooth structure and dental tissues with the respective stem cell populations. (**A**) The odontoblast niche is bordering dental pulp beneath the dentin with odontoblast processes projecting towards enamel. (**B**) Diverse cell populations are found in dental pulp, DPSCs, which can give rise to odontoblasts. (**C**) Cementocytes are residing in the lacunae of cellular cementum at the root apex with their cellular processes projecting towards the periodontal ligament.

The mature tooth is a complex organ consisting of non-vascularized hard tissues: enamel, dentin, and a soft vascularized innervated dental pulp. The dental pulp is closely associated with dentin and harbors odontoblasts, dental pulp stem cells (DPSCs), pericytes, and other cellular populations. Blood vessels penetrating the pulp nourish the resident cells, while nerves participate in the sensory information exchange between the pulp and oral environment (Figure 1B). In the case of excessive dental injury (e.g., deep caries), odontoblasts, their precursors, and DPCSs can be recruited from the dental pulp and participate in dentin repair [74]. The tooth is surrounded by the periodontal ligament, which is a complex attachment tissue harboring odontogenic stem cells [69,75], linking the tooth to the alveolar jawbone (Figure 1). The mature molar tooth macrostructure and microstructures of dental tissues containing cell niches are depicted in Figure 1. 

### 2.2. Signaling Pathways Modulating Hard Dental Tissue Generation

Many signaling cascades such as FGF, sonic hedgehog (Shh), transforming growth factors beta (TGF-β), BMPs, and Wnt/β-catenin are involved in the regulation of dentogenesis during development and adulthood [11,45,76,77,78]. Specific functions elicited by activation of these pathways are noted during distinct phases of dental tissue differentiation, some of which are beneficial for cell stemness and proliferation (FGF, Shh) while others such as Wnt, TGF-β, and BMPs act in postnatal differentiation phases and promote polarization, migration, and calcification [23,25,26,27,28,30,31,37,77,79,80]. Next to this, purinergic signaling function is gaining research attention in dental tissues metabolism [32,81,82]. Most ligands activate transcription factors such as runt-related transcription factor 2 (Runx2), osterix (Osx or Sp7), and extracellular signal-regulated kinase 1/2 (ERK1/2 or MEK1/2), which are central regulators of gene sets crucial for calcified tissues [33,34,35,83]. Epithelial–mesenchymal interactions are also involved in odontogenic and cementogenic differentiation [23,24,30,38,39].

#### 2.2.1. Amelogenesis 

Tooth enamel formation or amelogenesis is the process of tooth enamel generation by ameloblasts, during which ameloblasts move towards the enamel surface and secrete proteins such as amelogenin, ameloblastin, and enameling. These proteins serve as scaffolds for calcium and phosphorus ions to be deposited on, thus guiding hydroxyapatite crystals aggregates—the enamel rods—generation. In this process, amelogenin and amelotin phosphorylation appears to be essential for correct enamel rod formation/organization [84,85,86]. The scaffolds are later degraded by matrix proteases and ameloblasts undergo apoptosis, which makes enamel the most mineralized acellular tissue in the human body, consisting of 95% hydroxyapatite crystals and 5% organic matter and water by weight [10,11]. Enamel is subjected to wear and tear throughout life. However, unlike other mineralized tissues of the human body, enamel cannot be regenerated due to its acellular nature. Although several cell sources were shown to have amelogenic capacity including keratinocyte stem cells, epithelial cell rests of Malassez (ERM) from periodontal ligament, odontogenic oral epithelial stem cells (OEpSCs), adipose tissue-derived mesenchymal stem cells (AT-MSCs), and iPSCs [87,88,89,90,91,92].

Since ameloblasts undergo apoptosis upon fulfilling their function of enamel production, studies of amelogenesis rely on in vitro models such as murine immortalized ameloblast-lineage cell (ALC) line [15], organotypic cultures, or rodent models. Many discoveries regarding ligands, their downstream transcriptional factors and responsive genes expressing core enamel proteins and matrix metalloproteinases were done using the mentioned ALC line. Shh, which is one of the major ligands expressed in the enamel knot during tooth morphogenesis, was shown to have a direct effect on the expression of the major enamel matrix proteins amelogenin and ameloblastin. The upregulation of these proteins is mediated by an activated glioma-associated transcription factor (Gli1) in the presence of Shh [42,77]. Notably, Gli1 was proposed as a marker for selecting stem cells with the odontogenic potential for tooth regeneration [93,94]. Runx2 together with odontogenic ameloblast-associated protein (ODAM) regulates matrix metalloproteinase 20 (MMP20) expression, the key enamel matrix-degrading enzyme [43], and has an affinity for the *Wdr72* (gene coding for maturation-stage ameloblast-specific protein) promoter [44]. WDR72 is an intracellular protein abundant in ameloblasts during enamel maturation with a proposed function in amelogenin endocytosis [20]. 

Studies in dental organotypic cultures and transgenic mice also point out the importance of the mentioned pathways in dentogenesis. For example, Shh in combination with FGF8 was recognized as a stemness promoting ligans for ameloblast precursors (human skin fibroblasts) in a human-mouse chimeric tooth [87], while Runx2 was shown to have an affinity for the amelotin promoter and regulates its expression during the enamel maturation stage [95]. Regarding amelogenin turnover, a novel role of cytoplasmatic B-cell CLL/lymphoma 9 protein (Bcl9), its paralog B-cell lymphoma 9-like protein (Blc9l) and interaction partners Pygopus 1/2 (Pygo1/2) is proposed to play a role in amelogenin secretion [96].

Timely expression of β-catenin in dental tissues shapes tooth development by modulating various developmental signaling pathways, leading to the proper tooth number and morphology [45]. It was demonstrated in vitro that the β-catenin pathway, which is regulated by Wnt ligands, is involved in ameloblast polarity and motility [97]. Overactivation of the β-catenin pathway in the dental epithelium during the earliest stages of tooth development results in hyperdontia, and ablation—in tooth agenesis [98]—while, if overactive in postnatal ameloblasts, it causes poorly structured, softened enamel and its delayed formation [46]. Additionally, β-catenin overactivation downregulates enamel matrix metalloproteinases MMP20 and kallikrein 4 (Klk4), which are important in the removal of scaffolding proteins from maturing enamel [46]. An important regulator of Wnt/β-catenin pathway activity in ameloblasts is glycogen synthase kinase 3 beta (GSK3β) [99].

TGF-β superfamily ligands such as BMPs and TGF-βs are regulating enamel structural genes and matrix metalloproteinases expression. MMP20 in turn regulates TGF-β isoforms activity [47,100]. All three TGF-β isoforms induce Klk4 expression, while TGF-β1 and β2 induce amelotin expression [47]. TFG-β1 regulates Runx2 and its downstream target Wrd72 gene [44]. Thus, it appears that TGF-βs are key ligands involved in the regulation of enamel scaffolding protein removal and endocytosis during enamel mineralization. BMP knock-outs result in downregulated matrix proteins and metalloproteinase expressions. In detail, BMP2 knock-out reduced amelogenin, enamelin, MMP20, and Klk4 expression, similarly to double-knockout of BMP2 and -4, which resulted in a significant reduction of MMP20 and Klk4 in ameloblasts [21,22]. Metalloproteinase insufficiency is detrimental for the enamel structure since excessive protein content in enamel does not allow properly organized crystalline structure formation, making the enamel softer and less shear-resistant. 

**Figure 2:**
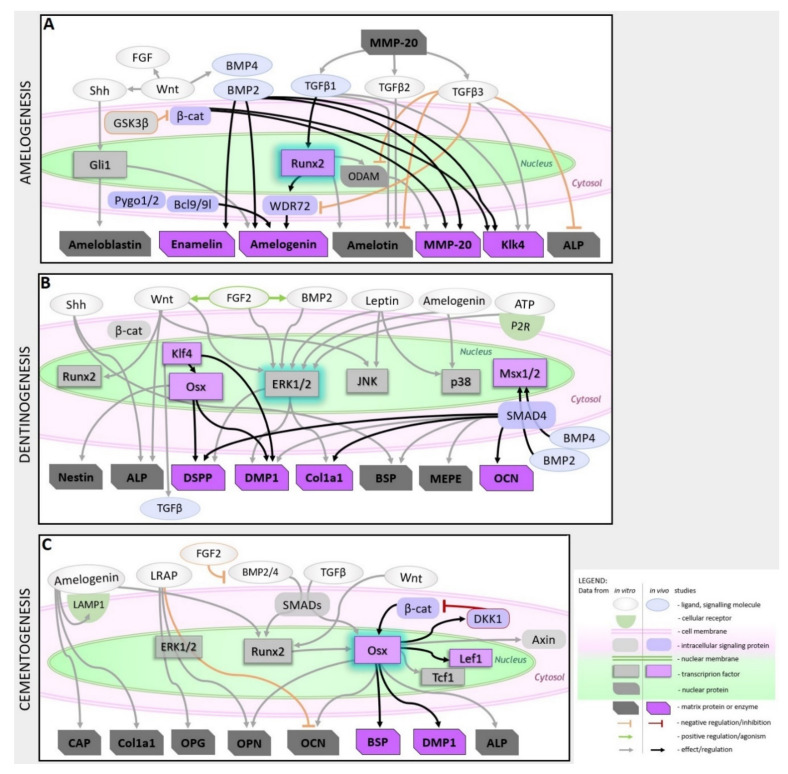
Major signaling cascades involved in amelogenesis, odontogenesis, and cementogenesis. (**A**) Signaling pathways modulating amelogenesis with TGF-β superfamily ligands (BMP2 and TGF-β1/2/3) playing the major role in matrix protein and metalloproteinases feedback-regulation and Runx2 being an important transcription factor. (**B**) Central signaling cascades of odontogenesis are depicted. The TGF-β superfamily ligands (BMP2/4 and TGFβs) regulate many odontogenic genes with ERK1/2 as convergence point and Klk4-Osx as important transcription factor tandem. (**C**) Major cementogenesis-related signaling cascades with Osx as the central transcription factor being regulated via Wnt/β-catenin in a feedback-loop. Ameloblast-derived products (LRAP and amelogenin) were shown modulate key cementogenic gene expression in vitro.

From the above-reviewed studies, it is evident that timely regulation of ligands known to be important for cell stemness maintenance and calcified tissue metabolism are the keys to structurally and morphologically correct enamel formation. Enamel integrity depends on proper enamel scaffolding protein deposition, phosphorylation state, and timely cleavage, which allow ameloblast migration and crystals deposition in an organized oriented pattern. The summary of the major signaling pathways involved in amelogenesis is schematized in Figure 2A and pathways modulators listed in Table 1. 

#### 2.2.2. Dentinogenesis

Dentin is an acellular calcified tissue consisting of 70% hydroxyapatite, 20% organic phase, and 10% water by weight. Dentin formation is executed by odontoblasts (or dentinoblasts), which are cells of mesenchymal origin. During dentinogenesis, odontoblasts migrate towards dental pulp and deposit collagen types I, III, and V, proteoglycans, and other matrix proteins, which provide the nucleation base for hydroxyapatite crystals. Besides scaffold-mediated mineralization, minerals precipitation and cell-derived matrix vesicles-driven mineralization occur during various stages of dentinogenesis [12]. After dentin synthesis is complete, odontoblasts remain beneath it with tiny cellular projections called odontoblast processes protruding into the microscopic channels in the dentin (Figure 1A). These projections are involved in detecting environmental stimuli (pH, cytokines, inflammatory mediators, and other signaling molecules) by odontoblasts, which can be mobilized for dentin regeneration in a case of damage. Thus, dentin possesses a limited capacity for regeneration [5,12,71,76]. Therefore, finding the appropriate cell source and differentiation strategy for dentin regeneration is of crucial importance. Thus far, dental pulp stem cells (DPSCs), stem cells from human exfoliated deciduous teeth (SHEDs), AT-MSCs, bone marrow-derived MSCs (BM-MSCs), and iPSCs have been shown to have the dentinogenic potential [25,80,81,90,101,102,103].

Shh is secreted by an epithelial cell layer, the zone of amelogenesis initiation, and serves as a paracrine differentiation signal for odontogenic cells [23,30]. It is later secreted by dentinoblasts during dentinogenesis and dental pulp stem cells (DPSCs), suggesting its autocrine function in odontogenic differentiation and dental pulp stem cell niche maintenance [30]. Amelogenin, secreted by ameloblasts, also participates in odontogenic differentiation of DPSCs by upregulating dentin sialophosphoprotein (DSPP) and dentin matrix acidic phosphoprotein 1 (DMP1) expression via the ERK1/2 and p38 pathways [104]. A similar effect could be achieved by the application of leptin: DSPP and DMP1 expression and ERK1/2, p38, and c-Jun N-terminal kinase (JNK) phosphorylation levels were markedly increased in leptin-treated DPCs [25,105]. Moreover, leptin application in the induced pulp cavity in rats leads to increased dentin formation during reparative dentinogenesis [106].

FGF exerts a time-dependent effect on dental-pulp derived odontoblast precursors. Transient exposure to FGF2 during the proliferation phase is beneficial for odontogenesis while no such effect is achieved upon constitutive FGF application until the maturation phase. FGF2 induces DSPP and DMP1 expression, which is also mediated via ERK1/2 pathway activation. Moreover, the agonistic effect on BMP2 and Wnt signaling during early odontogenesis were noted in cells treated with FGF2 [26,27].

BMP/TGF-β signaling is important during early odontogenesis, where it activates SMADs and regulates Msx-1/2 transcription factors expression, as well as in differentiated odontoblasts, for matrix gene expression [101,107,108,109]. BMP2 positively regulates odontogenic differentiation of stem cells from exfoliated deciduous teeth (SHEDs) by promoting the expression of DSPP, DMP1, and matrix extracellular phosphoglycoprotein (MEPE) [80]. BMP2 knock-out in dental mesenchyme results in dentin deposition and microstructure abnormalities indicating its pivotal non-redundant role in early dentinogenesis [28,107], while BMP2 together with BMP4 have redundant functions in mature odontoblasts where they regulate DSPP, DMP1, bone sialoprotein (BSP) and collagen type I alpha-1 (Col1a1) expression [108]. Smad4, the intracellular component downstream of BMP/TGF-β signaling, is also necessary for DSPP, Col1a1, and osteocalcin (OCN) expression and proper odontoblast maturation. If Smad4 is ablated, dentin formation is largely impaired and does not reach normal thickness in mice [29]. 

Wnt ligands are involved in odontoblast differentiation from mesenchymal precursor cells during the early stages of tooth development and later regulate dentin matrix deposition. It is proposed that at early stages of tooth development some Wnt ligands exert effects via the canonical Wnt/β-catenin signaling cascade and support odontoblast precursor cells stemness, while other Wnt ligands expressed at later developmental stages activate non-canonical pathways and promote the migration, proliferation, and mineralization of odontoblast precursors during dentinogenesis [31,83,110]. Experiments in vitro demonstrated that Wnt7b stimulates the expression of Runx2 and the key dentin matrix proteins DSPP, DMP1, and Col1a1 via ERK1/2-mediated activation during dentinogenesis [83]. Wnt7b can activate canonical Wnt/β-catenin, but also the JNK cascade, thus promoting cellular migration and odontogenic differentiation [31]. Notably, activation of Wnt/β-catenin signaling by inhibition of GSK3β is beneficial for reparative dentine formation during cavity repair [111].

Purinergic signaling mediated by adenosine receptors (P1 receptors, ARs) and purine receptors (P2X and P2Y) was also shown to play an important role in odontogenic differentiation of human DPSCs. P2 receptor activation by ATP promotes the expression of DSPP, DMP1 and mineralization of DPSCs via rapid phosphorylation of ERK1/2 [32]. Treatment of DPSCs with P1 receptor agonists in combination with ATP further improved odontogenesis by contributing to the upregulation of DSPP (mediated by A2BR and A3R) and DMP1 (via A1R and A2BR) and increased mineralization (via A1R and A2BR) [81]. Intracellular molecular events of P1 and P2 receptors agonistic action remain to be elucidated, but ERK1/2 is likely involved, at least partially, in the purinergic receptor-mediated odontogenic differentiation of DPCs as is the case with several other differentiations regulated by purinergic signaling.

Aside from the importance of activation of ERK1/2 and its downstream targets resulting in the expression of key dentin matrix genes, Tao and colleagues outlined Krüppel-like factor 4 (Klf4) as a major transcription factor regulating odontogenesis [33]. Klf4 induces TGF-β secretion, which together with BMPs positively regulates DMP1, the major dentin matrix protein expression. Moreover, Klf4 regulates odontogenesis-related gene expression temporally by interacting with histone deacetylase 3 (HDAC3) during early phases of odontoblastogenesis where it represses the expression of *osterix* and *DSPP*, while at later stages, when paired up with P300, it promotes their expression [33]. Osterix is a master-regulator of many structural genes of dentin and also of odontoblasts including DSPP, DMP1, nestin, and alkaline phosphatase (ALP) [34].

Studies regarding odontoblast differentiation outline the importance of signaling pathways and their interactions alike noted to be important for ameloblast differentiation with ERK1/2 being a convergence point for several signaling cascades involved in odontogenic differentiation of dental mesenchymal cells. Recently identified Klk-Osx transcriptional tandem, p38 and JNK are important in dentin structural genes regulation and odontoblast function (Figure 2B). Several dentinogenesis-promoting molecules (listed in Table 1) were already tested in vivo and shown promising results.

#### 2.2.3. Cementogenesis 

Cementum, a thin calcified avascular tissue between dentin and periodontal ligament, is produced by cementoblasts. Cementum contains collagen type I, bone sialoprotein, osteopontin, glycoproteins and proteoglycans arranged in a fibrous network with hydroxyapatite deposits. Various types of cementum are present in distinct regions of mature tooth roots: thin acellular cementum is deposited around the cervical tooth area and below, while thick cementum with entrapped cementocytes and their processes penetrating cementum locates at the root apexes (Figure 1C). Histological studies also indicate that a thin layer of dense acellular cementum lies beneath the cellular cementum at the root apex and plays an important role in cementum mineral metabolism. The cementum volume is enlarging over the lifespan and is not subjected to remodeling such as bones. Cementoblast precursors are present in the periodontal ligament and can be mobilized for cementum regeneration if needed [13,41,112]. Ex vivo, cementoblasts can be generated from periodontal ligament stem cells (PDLSCs), dental follicle stem cells (DFSCs), and iPSCs [75,113,114].

By analogy with dentinogenesis, TGF-β, and BMPs, Wnt and ameloblast-derived factors regulate cementum structural matrix protein expression. The central transcription factor of cementogenesis is Osx, which is activated by Wnt and TGF-β/BMP signaling. Osx is abundantly expressed in cementoblasts and cementocytes during cementum deposition, where it regulates DMP1, BSP, OCN, and ALP expression. It is proposed that Osx regulates cementogenic differentiation, while it inhibits cementoblast proliferation [35,115]. Stabilization of β-catenin leads to increased cementum formation via the upregulation of Osx, which is achieved by β-catenin binding to the Osx promoter, thus pointing to the direct regulation of Osx by β-catenin [36]. Additionally, Osx regulates the expression of dickkopf-related protein 1 (DKK1), an antagonist of β-catenin, and the transcription factors T-cell factor 1 (Tcf1) and lymphoid enhancer-binding factor 1 (Lef1), which together with β-catenin form a transcription initiation complex with β-catenin in the cell nucleus. It is therefore evident that cross-regulation of β-catenin and Osx plays a central role in cementogenesis [36,115]. 

In addition to Wnt/β-catenin regulation, Osx is regulated via the TGF-β/Smad axis, as Smad3 plays an important role in *Osx* gene expression during cementogenesis [37]. BMP2 and -4 likewise regulate Osx expression via a BMP-Smad-Runx2 cascade, but also Runx2-independently [34]. Despite the suggested beneficial role of Wnt/β-catenin in cementogenesis, another point of view has been expressed, according to which excess Wnt may inhibit cementogenesis under normoxic conditions, while hypoxia reverses this effect [24]. BMP2/4 signaling, which promotes cementogenesis in several ways, is negatively regulated by FGF2 in a concentration-dependent manner. This has been shown in periodontal ligament cells undergoing cementogenesis, thus implying that FGF2 is not beneficial for differentiation, but is important for cellular stemness [75]. This is in line with similar results in amelogenesis or very early stages of odontogenic differentiation [27,75,87]. Contrarily, in vivo, local FGF2 infusion was shown to promote cementum formation during periodontal injury regeneration by recruiting, enhancing and accelerating the proliferation of endogenous cemento/ostogenic cells [116].

The enamel-derived signaling components, amelogenin and its alternatively-spliced isoforms, regulate cementogenesis by modulating the expression of various matrix proteins. Full-length amelogenin application induced the expression of osteopontin (OPN), cementum attachment protein (CAP), OCN, Cola1, BSP, DMP1, and ALP mRNA; upregulated OPN and Col1a1 proteins; and improved the mineralization of an immortalized mouse cementoblast cell line (OCCM-30). Moreover, amelogenin positively regulated its putative receptor lysosome-associated membrane glycoprotein 1 (LAMP1) in murine dental follicle cells and OCCM-30 cells, thus confirming its role as an important ligand regulating cementogenesis [38,39]. Amelogenin derivates, such as leucine-rich amelogenin peptide (LRAP), modulate gene expression in a slightly different manner: LRAP inhibited OCN expression, while promoted OPN and osteoprotegerin (OPG) expression in a dose-dependent manner and had a negative effect on cementoblast mineralization. The effects are probably mediated through the ERK1/2 pathway since ERK inhibition annuls the LRAP effects [79]. 

Similar to dentin and dentinogenesis, cementogenesis has a central transcription factor: Osx, which regulates cementogenesis-specific gene expression. Besides Osx, Runx2, and ERK1/2 are involved in cementogenic differentiation. In addition, ameloblast-derived proteins are important ligands positively regulating cementum matrix-associated gene expression (Figure 2C). Modulators of herein discussed cementogenic pathways are listed in Table 1. 

Gained knowledge about molecular cues shaping dental tissue genesis may help to establish novel stem cell selection, culture, and differentiation methods and develop functionalized scaffolds and biomaterials, which will support and promote amelogenic, dentinogenic, and cementogenic differentiation in vitro. Thus, it will approximate the era of dental tissues regeneration using most suitable odontogenic cells with adequately functionalized biomaterials. 

## 3. Scaffolds and Drug Release Materials for Tooth Regeneration

### 3.1. Scaffolds for Enamel, Dentin, and Cementum Regeneration

Scaffolds and biomaterials are essential components in dental tissue regeneration since they can be used as a template for tissue regeneration by serving as a site of attachment for the regenerative cells from the surrounding tissues or act as a delivery platform for implantable odontogenic cells with the ability to differentiate towards the desired cell type [122,123]. Additionally, the scaffold material may be used as a delivery platform for bioactive molecules such as drugs or proteins (especially growth factors) that further enhance the regenerative potential [60,61,63,124].

In general, scaffold materials used in tissue regeneration need to be readily available and meet criteria such as biocompatibility and biodegradability without any toxic metabolites. In the case of scaffolds for tooth regeneration, biomaterials are subjected to the challenging environment of the oral cavity—including mechanical forces due to mastication, the presence of microorganisms, and varying conditions regarding temperature and pH. The intended biomaterial has to face these challenges without limitations in its biocompatibility [125]. Since it is generally intended to mimic the native extracellular matrix by using biomaterials, properties besides biocompatibility are imposed by the tissue which should be regenerated. Thus, in the case of scaffold materials for dental tissue engineering, the used material systems differ greatly depending on whether enamel, endodontic, or periodontic tissue is intended to be regenerated. Categories for biomaterials used in tooth regeneration are natural organic, synthetic organic materials, or inorganic materials [126]. Natural organic materials involve peptides such as collagen or gelatin and polysaccharides such as chitosan, alginate, or agarose. Frequently used synthetic organic materials are poly(lactic acid) (PLA), poly(glycolic acid) (PGA), poly(lactic-co-glycolic acid) (PLGA), and poly(caprolactone) (PCL), while commonly used inorganic materials are bioactive glasses or calcium phosphates such as hydroxyapatite (HA), β-tricalcium phosphate (TCP), and cementitious systems of calcium phosphate (CPC) or calcium silicate (e.g., mineral trioxide aggregate, MTA). Polymeric materials often lack mechanical and biological properties but are able to establish three-dimensional porous structures, thereby providing a highly hydrated matrix in vivo that facilitates the transport of nutrients, anabolites, and catabolites. In turn, inorganic biomaterials used in tissue engineering often comprise preferable biological properties but have disadvantages such as brittleness and lacking in the supply of nutrients. Thus, composite materials comprising both organic and inorganic constituents gain increasing interest in recent years due to their inherent combination of the desirable properties of the single components [127]. In the following subsection, the challenges, approaches, and recent studies for the targeted and scaffold-assisted regeneration of enamel, dentin, and cementum are presented. Injectable biomaterials are a central and highly desirable class in the context of dental regeneration, but are not extensively reviewed here due to the very recent and detailed publication of a distinct review on this topic by Haugen and coauthors [128].

#### 3.1.1. Enamel Formation

The main challenge in the regeneration of enamel is its acellular nature. Enamel forming ameloblasts go through apoptosis when amelogenesis is finalized and the in vitro culture of ameloblasts is yet unestablished in a scale needed for appropriate tissue regeneration [129]. Furthermore, although the synthesis of hydroxyapatites is widely investigated, attempts to model the unique assembling of HA-crystals in enamel were not yet successful [130]. Thus, many recently published studies follow a biomimetic approach by using amelogenin, peptide fragments of amelogenin, or various synthetic peptides as a template matrix to mimic the spatiotemporal environment for the deposition of enamel.

Recently, Zheng et al. used a peptide consisting of eight repetitive sequences of aspartate-serine-serine (8DSS) as a biomimetic template for enamel remineralization in an in vivo model. Their results indicate that 8DSS peptides serves as both inhibitor of further enamel demineralization and promoter of remineralization by entrapping calcium and phosphate from the surrounding medium. As a result, mineral density and enamel volume increased to a comparable extent as with a fluoride treatment [131]. Treating enamel surface with an elastin-like polypeptide (ELP) functionalized with glutamic acid residues to dissolve calcium and phosphate due to its acidic properties leads to a matrix consisting of ELP and amorphous calcium phosphate (ACP). After immersing the specimen in simulated oral fluid, a dense layer of highly orientated apatite nanorods is formed from the matrix with mechanical properties close to natural enamel and high chemical stability against acidic impacts [132]. The properties of poly(amidoamine) (PAMAM) dendrimers can be tailored by modification of their functional surface groups. Accordingly, the effect of amino-, carboxyl-, and alcohol-terminal groups has recently been studied in vitro. The results show that the electrostatic interactions between biomaterial and enamel surface affect the remineralization process. PAMAM-NH2, exhibiting interactions between pro-cationic amino groups and negatively charged enamel surface, shows the best results, followed by PAMAM-COOH due to interactions between carboxylate residues and calcium cations in hydroxyapatite, while neutral PAMAM-OH was not effective [133]. Additionally, Gao et al. evaluated the performance of amorphous calcium phosphate loaded PAMAM-dendrimers functionalized with an SN15 peptide sequence, which is known for its good adsorption on hydroxyapatite, for the use as adhesive in resin-based approaches of caries lesion treatments and achieved 90% higher remineralization compared to control [134].

#### 3.1.2. Dentin Formation

Dentin regeneration is most often related to a treatment of the dentin-pulp complex. Since pulp vitality is essential for tooth homeostasis and stability, strategies to maintain this vitality are highly desirable. Presently, pulp capping is the main therapy maintaining the pulp vitality but is frequently accompanied by irreversible pulp inflammation and reinfections [16]. Thus, innovative approaches and biomaterials for the regeneration of the pulp–dentin complex are highly desirable. 

In classical endodontic therapy via apexification, the pulp space is initially cleared and sealed with calcium hydroxide or MTA to induce a hard-tissue formation at the apical area that is used as a barrier for a permanent root filling material. Since this procedure does not promote further root development, root canal walls remain thin and fragile, leading to teeth that are prone to further issues [135]. To overcome these limitations, regenerative endodontic therapies including revascularization are being developed. Here, bleeding is induced to fill the endodontic canal and form an autologous blood clot that serves as a scaffold homing matrix proteins, (stem) cells, and growth factors, which consequently leads to the regeneration of the pulp–dentin complex due to root development, apical closure, and maintenance of the tooth vitality [17,136]. However, due to the presence of mesenchymal stem cells in the infiltrating blood, the generated tissue is more bone-like mixed with connective tissue instead of the desired pulp–dentin complex [137].

Recently, Mandakhbayar and colleagues used strontium-free and strontium-containing nanobioactive glass cement in a pulp capping approach to evaluate their potential to regenerate the pulp–dentin complex in vitro and in vivo [138]. The nanobiocement based on mesoporous calcium silicate nanobioactive glasses showed a fast release of Ca-, Sr-, and Si-ions, which are known for their bioactive properties in hard-tissue regeneration; promoted the odontogenesis of DPSCs in vitro; and showed promising results in vivo, especially for Sr-containing biomaterials [138]. Boron-modified bioactive glass nanoparticles were embedded in an organic matrix of cellulose acetate, oxidized pullulan, and gelatin by Moonesi-Rad and associates to build a dentin-like construct by freeze-drying and subsequent mold pressing [139]. The composite material induced the enhanced deposition of a calcium phosphate layer after immersion in simulated body fluid. Moreover, cell culture studies using DPSCs indicated the promotive effects of boron-modified bioactive glasses on attachment, migration, and odontogenic differentiation [139]. In a classical ternary system comprising an injectable collagen scaffold, DPSCs, and growth factors, Pankajakshan and coworkers evaluated the effect of mechanical properties of the collagen matrix [140]. Via concentric injection, the authors created a scaffold with an inner section of lower stiffness, which is covered with an outer section of higher stiffness to mimic the mechanical properties of the natural pulp–dentin complex. Additionally, they loaded the softer scaffold material with proangiogenetic vascular endothelial growth factor (VEGF) and the stiffer scaffold material with BMP2 to enhance the site-specific endothelial or odontogenetic differentiation of DPSCs, respectively. The results show that the stiffness of the materials regulates the direction of DPSCs differentiation. This effect is further enhanced by the loading of the collagen matrices with VEGF or BMP2, respectively [140].

#### 3.1.3. Cementum Formation

Cementum regeneration is closely related to the treatment of the periodontal complex comprised of alveolar bone, periodontal ligament, gingiva, and cementum (Figure 1). Besides the structural support a scaffold material provides to the affected tissue, scaffolds used for regeneration of the periodontal complex are often used as a delivery vehicle for various bioactive compounds such as proteins, growth factors, or gene vectors to favor the regenerative process and induce the recruitment and homing of endogenous stem cells from surrounding tissues. The development of multicompartment scaffolds aims to meet the diverse challenges of the different tissues to be regenerated in periodontal defects in a single scaffold [141]. Additionally, besides synthetic scaffolds, cell-based scaffolds such as cell sheets are part of current research. In this approach, cell types that are relevant for the periodontal regeneration are cultivated in vitro extensively, until strong cell–cell interactions are established and an extracellular matrix has formed, thus allowing transplantation of the cell sheet as a scaffold-like material [142].

Recently, Fakheran and peers evaluated the regenerative potential of Retro MTA, a calcium silicate cement, in combination with tricalcium phosphate in vivo and showed that newly formed bone and cementum was significantly higher than in the untreated control group. Moreover, the poor biodegradation rate of MTA is improved due to the combination with biodegradable TCP [143]. In a preclinical study to treat periodontal defects in dogs, Wei et al. used an inorganic calcium phosphate-based scaffold material loaded with BMP2 [144]. The CaP-based biomaterial alone leads to a significantly increased regeneration of mineralized tissue as well as to an improved attachment of the teeth to the surrounding tissue compared to untreated control and a deproteinized bovine bone mineral that serves as commercial control. When loaded with BMP2, these positive results could even be improved two- and three-fold regarding height and area of the remineralized tissues, respectively. Noteworthy, the encapsulated BMP2 had a greater impact on osteogenesis than on cementogenesis [144]. Following the multicompartment-scaffold approach, Wang and collaborators applied a bilayered material containing growth factors. The hybrid material containing an FGF2-loaded propylene-glycol alginate gel coating the root surface for ligament regeneration and a BMP2-loaded (PLGA)/calcium phosphate cement for periodontal regeneration was tested in vivo with non-human primates. Following a promising study in rodents, the authors reported significantly enhanced regeneration of cementum and periodontal ligament and a high vascularization of the newly formed periodontal ligament (PDL), thereby confirming the positive results of the previous study [145,146].

Vaquette el al. developed bilayered scaffold materials based on polycaprolactone and combined them with cell sheets: while a fibrous three-dimensional compartment with macropores should favor alveolar bone regeneration, a flexible porous membrane aims at delivering the cell sheet and regenerates the periodontal ligament [147]. In their study, the authors evaluated the in vivo regenerative potential of the hybrid materials with different cell types forming the cell sheet, namely gingival cells, periodontal ligament cells (PDLCs), and bone marrow-derived mesenchymal stem cells (BM-MSCs). Results from histomorphometry and micro-computed tomography (µ-CT) show that scaffolds containing BM-MSCs and PDLCs had greater regenerative potential due to superior new bone and cementum formation compared to the scaffolds containing gingival cell sheets. However, the regenerative potential of scaffolds containing BM-MSCs and PDLCs did not differ significantly compared to the performance of the non-cellularized control scaffold. Thus, the biphasic scaffold alone is also a promising candidate for further studies [147]. Table 2 summarizes recently published studies emphasizing regenerative approaches of enamel, dentin, and cementum.

### 3.2. Drug Release Systems Useful in Tissue Engineering—To be Adapted to Tooth Engineering

As discussed in the previous section, whole tooth regeneration is one of the most challenging fields in regenerative medicine—also regarding drug release aspects. In stem cell-based approaches, a cocktail of different drugs would be required to tightly tailor the differentiation of the corresponding cells involved in amelogenesis, dentinogenesis, and cementogenesis, respectively. This means that, besides appropriate scaffolds, compounds have to be developed for drug encapsulation and controlled release of those substances involved during tooth formation (such as growth factors and receptor ligands, as listed in Figure 2). Thus far, drug release approaches in tooth regeneration are mainly restricted to the delivery of antibiotics to avoid inflammation [66].

In analogy to other tissues and organs engineered using stem cell-based approaches, the drug delivery systems (DDS) are mainly classified into the following release mechanisms: diffusion through water-filled pores; diffusion through the polymer; osmotic pumping; and erosion [148]. In the past two decades, novel release materials have been designed and prepared that could be classified into the following three groups: (a) polymer-based systems; (b) ceramics-based systems; and (c) hybrid systems (e.g., organic/inorganic and polymer/ceramic) [62,149]. Many of them are prepared as nanomaterials (e.g., spheres, capsules, and rods) [64].

To develop a DDS that allows kinetically controlled release of drugs supporting the required stem cell differentiation processes, a variety of material characteristics would have to be considered. Parameters that influence the release behavior of polymer-based release materials include the following: molecular weight (number and weight average, respectively, M_n_/M_w_) and corresponding polydispersity index (PI), number and nature of end-groups, and the polymer morphology mainly determined by the monomer 3D structure (amorphous and crystalline/semi-crystalline with the degree of crystallinity). All of them are able to influence the size and shape, as well as density and porosity of the entire DDS that includes the encapsulated drugs. In addition, the active substance (drug) itself influences the release kinetics via interaction with the encapsulation material. Thus, the drug hydrophilicity/hydrophobicity (resulting from chemical composition, functional groups, hydrogen bonds, etc.) is one of the most limiting aspects, as well as its ability to act as surfactants or plasticizer which would interfere with the release mechanism. Huang et al. comprehensively reviewed the release mechanisms discovered within the last five years, including drugs for tooth regeneration [65]. Most recent developments include tunable conductive polymers to be used for controlled delivery [150]. As stated in Section 3.1, in tooth regeneration, drugs (such as growth factors and FGF-2) are usually simply added to the scaffold material—not yet encapsulated and released from tailored delivery materials [14,19,66,67,151,152,153,154,155,156,157]. Recently, Moon et al. reported a study using nitric oxide release to support the pulp–dentin regeneration [158]. However, in this case, release kinetics cannot be controlled or adjusted to the differentiation processes of the corresponding cells. Very few studies reported the application of specific drug encapsulation materials, mainly using hydrogels [63,64,65,159,160,161]. Hydrogels can easily be prepared using natural and artificial polymers (sometimes a combination of both classes). One of the most prominent groups of hydrogels is based on polysaccharides [149,162,163]. Furthermore, other polymers such as polyvinyl alcohol (PVA), polylactic acid and polyglycolic acid (PGA), polyacrylic acid (PAA), and polyethylene glycol (PEG) are intensively studied regarding their ability to form hydrogels used for controlled delivery [160,164]. Hydrogels offer various advantages; most importantly, they are tunable in their chemical structure resulting in controlled degradability. In a comprehensive review, Li et al. discussed various multiscale release kinetic mechanisms of hydrogels and classified them according to the structural interactions. Thus, the kinetics are significantly determined by the hydrogel mesh size, network degradation, swelling, and mechanical deformation. In addition, kinetics depend on various interactions of the hydrogel components such as conjugation, electrostatic interaction, and hydrophobic association [164]. 

For hard tissue such as bone, our group could recently show that it is possible to guide osteogenesis via purinergic receptor ligand release. Osteogenesis of mesenchymal stem cells is influenced by various purinergic receptors (P1, P2X, and P2Y) [122,124,165,166,167,168]. Thus, a release of specific agonists and/or antagonists enables tailoring of the corresponding receptor up- or downregulation. Furthermore, besides osteogenesis, purinergic receptors are also involved in angiogenesis—a process also required during tooth regeneration [68,169,170].

**Figure 3:**
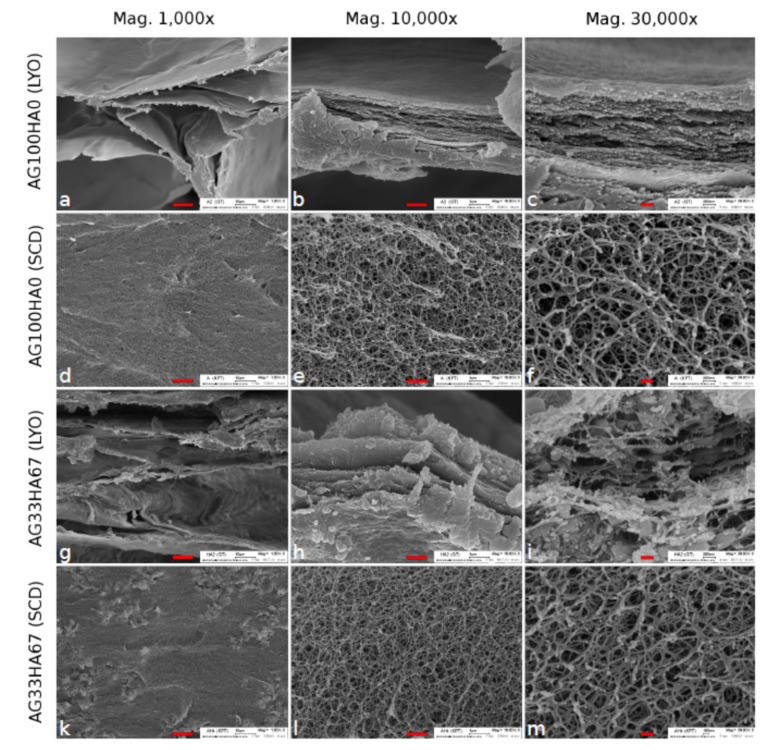
SEM images of agarose lyophilized (LYO) (**a**–**c**) and supercritically-dried (SCD) (**d**–**f**) and agarose/hydroxyapatite (33/76 w%) composite LYO (**g**–**i**) and SCD (**k**–**m**) at three different magnifications. The scale bars are 10 μm (left), 1 μm (middle), and 0.2 μm (right), respectively. Reproduced from Witzler et al., 2019 [171]. Open Access Copyright Permission (Creative Commons CC BY license).

In a recently published paper, we reported the synthesis and testing of novel hybrid release materials based on hydroxyapatite and agarose used to improve the release kinetics of drugs applied for guided osteogenesis [171]. Scanning electron microscopy (SEM) revealed details regarding the influence of the drying treatment: lyophilized (LYO) versus supercritically-dried (SCD) gels were tested and compared. As shown in Figure 3, SEM confirmed a homogeneous distribution of the elements involved in the hybrid (carbon, calcium, and phosphorus). In addition to SEM, energy-dispersive X-ray spectroscopy (EDX) results are given in [171]).

**Figure 4:**
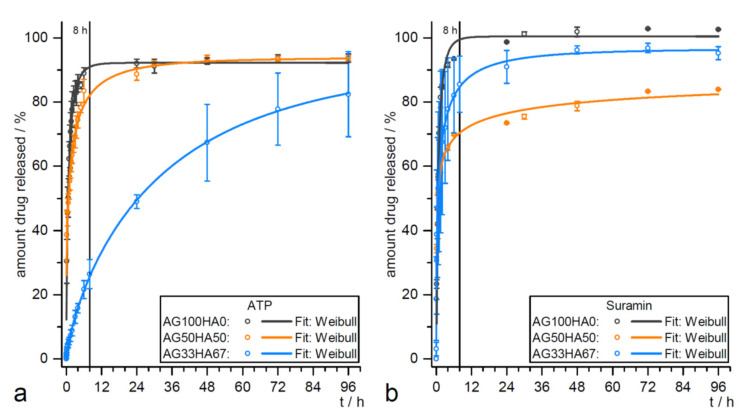
Release data of (**a**) adenosin triphosphate (ATP) and (**b**) suramin from agarose/hydroxyapatite (AG100HA0) (black), AG50HA50 (orange), and AG33HA67 (blue) scaffolds. Data fit: Weibull equation. Reproduced from Witzler et al., 2019 [171]. Open Access Copyright Permission (Creative Commons CC BY license).

Hitherto, hybrid systems are mainly studied as release materials for hard tissue regeneration [67]. Here, sustained delivery is required for guided stem cell differentiation, a burst release is favorable to achieve anti-inflammatory and antibacterial effects. Since both processes are also relevant in tooth formation, hybrid materials would be promising candidates to be investigated as release materials to improve cascades, as shown in Figure 2. In previous studies, the HA/agarose hybrids were loaded with model drug compounds for guided differentiation of MSCs. Different release kinetic models were evaluated for adenosine 5′-triphosphate (ATP) and suramin (Figure 4) [171]. Although both drugs are highly water-soluble, the release could be slowed to four days, which is significantly longer than comparable systems reported in the literature [172].

Future efforts should be directed toward the development of tailored drug loading and/or encapsulation materials to be used for the controlled release of bioactive substances during tooth formation [157,173]. As shown in Figure 2 and Table 1, there are various signaling molecules and corresponding activators and suppressor molecules involved in the formation of enamel, dentin, and cementum. For a number of these substances, loading and controlled-release from non-cytotoxic materials already exist, as shown in Table 3. Release materials mainly consist of natural or artificial polymers, but also hybrids composed of organic and inorganic components. The focuses of the studies are release kinetics and corresponding mechanisms. However, some drugs are being successfully applied in vivo. 

In detail, a sequential and on-demand release of multiple drugs (signaling molecules, activators, and suppressors) would be required to control and guide the signaling cascades of amelogenesis, odontogenesis, and cementogenesis [164]. Moreover, on-demand release systems usually require specific stimuli as reported for example for conductive polymer-based delivery devices [150]. Finally, theoretical modeling could provide a more fundamental understanding of release kinetics [189]. 

## 4. Whole Tooth Regeneration

The regeneration of a whole tooth as an organ replacement therapy is considered to be the ultimate goal of regenerative dentistry. For patients, this therapeutic option could represent a dream for the replacement of decayed or lost teeth to overcome prosthodontic or implantology treatment using artificial replacements. Whole-tooth generation could be performed as a hybrid strategy where, e.g., biologically created tissue compartments such as the periodontal ligament or a tooth crown would be combined with a metallic or ceramic implant or where a biological regenerated tooth root (“bio-root”) would be combined with a prosthetic crown (see, e.g., [190,191,192]). In the following years, efforts in creating a whole tooth from only cells and tissues (“bio-tooth”) will be very likely in the focus. However, despite all efforts and achieved results in basic and translational research, this approach is still challenging [48,58,69,193,194].

### 4.1. Reactivation the Odontogenic Potency

On the background of teeth evolution, a genetic approach to generate whole teeth may be an option in the far future. Teethed fishes, reptiles, or amphibians are polyphyodonty, which means that several tooth generations can be formed and erupted. This highly regenerative capacity was reduced during evolution. In mammals, many species including human are only diphyodont with the capacity to form a second dentition or even monophyodont such as the mouse [71,72,195]. Revitalizing the odontogenic potency for the lost tooth regeneration capacity may be an interesting approach to induce tooth formation in vivo in the adult. One prerequisite for tooth replacement is the existence of a successional dental lamina (SDL) carrying the capacity for inducing odontogenesis. Even in monophyodont animals, rudimentary SDL has been identified. In addition, in the human species, rudimentary laminae are preserved, which might be responsible for a third dentition but this, however, has been observed very seldomly. On a molecular level, tooth replacement is regulated by signaling pathways [71]. For example, in alligators or snakes, stem cells in the SDL express Sox2, which is initiated by the Wnt/β-catenin pathway an interacts with BMP signaling [195]. Dysregulation of Wnt-signaling is discussed to be important for the de-activation of rudimentary SDL as it occurs in the mouse. Therefore, the revitalization by stabilizing Wnt signaling by application of appropriate factors or genes could be a strategy for the induction of re-growing teeth in the future [195,196].

### 4.2. Tissue Recombination Approaches

The basic principle of this “classical” approach is to mimic the natural development and formation of a tooth and to recapitulate the signaling cascades regulating tissue interactions during odontogenesis. For over a hundred years, progress has been made in understanding tooth development in different species including human, identifying tissue interactions and factors involved on the morphological, cellular and molecular levels [18,58,65,71,193,195,197]. Classical tissue recombination experiments undertaken in developmental biology research have shown that mouse embryonic tooth germs can be dissociated and later re-aggregated. After temporary ectopic grafting of these cell aggregates, e.g., into the anterior eye chamber, subcutaneously, or under the renal capsule, tooth-like organs with mineralized tissues (dentin and enamel) could be grown (e.g., [198]). This method has been improved in the last years by using collagen drops for the organoid culture of 5–7 days or seeding the re-aggregated germ cells on biodegradable polymers [199,200,201]. The final goal of these experiments was to implant the constructs into the jaws of postnatal animals to generate a whole “bio-tooth”. In line with this cultured rat tooth, bud cells seeded onto biodegradable scaffolds for 12 weeks formed tooth-like crowns consisting of pulp, dentin, enamel, and periodontal ligament after implantation into rat jaws [200]. 

A breakthrough came with experiments of the group of Ikeda, who could demonstrate that the implantation of re-aggregated autogenous germ cells into the extraction socket of pigs succeeded in the formation, development, and eruption of teeth, which could be brought into occlusion and fulfilled all functions of normal teeth [50]. Over half (56%) of the implanted constructs had erupted. Later, it was also possible to create a unit of a regrown tooth with surrounding alveolar bone [202]. Whole-tooth restoration using autologous bioengineered tooth germ transplantation was also successful in canines [51]. An allogeneic approach was undertaken by Wu and colleagues, who transplanted re-associated tooth germs into the jawbones of minipigs [203]. A xenogeneic approach was published by Wang and co-workers in 2018 [52]. Cells from unerupted deciduous molar germs of pigs were recombined and transplanted first in mouse renal capsules and finally in jawbones. However, problems are caused by the limited sources of tooth germ cells and risks of immune rejection when using allogeneic or xenogeneic cells. In humans, there are many hindrances, e.g., that tooth germs may not be easily accessible, but also ethical and legal constraints must be considered. An alternative could be the use of adult stem cells (see Section 4.2) or of iPSCs [53,54]. 

Different types of adult dental stem cells, e.g., from the pulp, or differentiated orofacial cells, e.g., from the gingiva, can be used as sources to create iPSCs with a similar epigenetic pattern. These cells show the ability to differentiate into epithelial or mesenchymal tooth germ cells [58,92]. Cai and co-workers generated iPS cells from cells out of human urine, which were differentiated to epithelial sheets and recombined with embryonic mouse dental mesenchyme [91]. Tooth-like structures were generated in which the epithelial cells differentiated into enamel-secreting ameloblasts. The formation of enamel, the hardest tissue of the body (see Section 2.2.1), is an important step in generating whole teeth, but also would be of importance for repair or regeneration of enamel loss in conservative dentistry. Thus, it is of major interest to find tissue sources able to generate dental epithelial cells which can be differentiated into enamel-secreting ameloblasts. Aside from iPSCs, examples for this are epithelial cells from the skin or gingiva as well as epithelial rests of Malassez, which can be found in the PDL, co-culture of these cells with different types of dental mesenchymal cells can lead to ameloblast differentiation or even formation of enamel-like structures [58,87,89].

### 4.3. Adult Stem Cell Approaches

The optimal method to create whole teeth would be the use of autogenous dental cells from patients demanding tooth regeneration. For whole tooth bioengineering, different strategies in the application of these cells have been developed. One idea was to combine adult stem cells with cells of the progenitor cells of embryonic tooth. Adult stem cells should have an odontogenic competence and should function as a “tooth inducer” when combined with mesenchymal cells or they should express a dental mesenchymal competence when combined with dental epithelium. Already in 2002, Young et al. cultured cells obtained from unerupted porcine tooth buds [199]. The aggregates were grown on biodegradable scaffolds in vitro or transplanted. This led to the formation of a primitive tooth crown with pulp, dentin, and enamel formation. Later, similar bioengineered tooth-like structures could be obtained by using rat and human cells [204,205]. In 2004, Ohazama and colleagues used non-dental adult MSCs in combination with inductive embryonic dental epithelium first transplanted under the renal capsule and transplanted them in adult jaws. Tooth formation including root occurred and the teeth erupted. In addition, bone was induced [206]. Adipose-derived MSCs alone were able to generate tooth bud-like structures in vitro [90]. Human gingival epithelial cells were used by Volponi Angelova and associates and combined with embryonic mouse tooth mesenchyme, which yielded an entire tooth outside of an embryo [207]. 

However, for all these experiments, relatively large amounts of adult cell populations were necessary that should be able to retain any odontogenic potential and, in addition, a large number of embryonic cells was needed as well. In a case of embryonic mouse tooth mesenchyme, a minimum cell number of 4 × 10^4^ to 4 × 10^5^ was sufficient according to the experiments of Hu et al. (2006) [208]. Therefore, to do so, cells from multiple embryos must be harvested. Another problem is the loss of the inductive capacity already after 24–48 h in culture, which makes the in vitro expansion of these cells using standard methods impossible [209]. This phenomenon can be explained by the fact that mesenchymal stem cells lose their dense packaging formed by cellular condensation and thus their linked cell contacts, which is a prerequisite for an inductive capacity in vivo. Ongoing research focuses therefore also on how an odontogenic potential can be maintained in vitro [194]. 3D micro-culture systems such as the hanging drop method in liquid media may allow the preservation of such signals. However, many cells are necessary for these methods [210]. Gene expression studies must be undertaken to identify signaling factors, which are lost in 2D cell cultures. In a study using postnatal dental pulp stem cells, Yang and collaborators could obtain “a rescue” in cultured cells due to the combination with uncultured mesenchymal tooth germ cells [57]. This rescue or community effect is responsible for the reactivation of inductive signals. Forthcoming, iPS cells (see Section 4.2) may be an appropriate cell substitute to overcome these biological problems. 

In the future, research will presumably focus on using adult stem cells from dental and non-dental sources to test recombination or co-culturing for their effects on tooth development. Zhang and coworkers optimized such a method by recombinant 3D-tissue engineering of intact dental tissues and cell suspensions from postnatal porcine teeth and human third molars [211]. After osteogenic culturing and subcutaneous transplantation in athymic nude rat hosts, tooth-like constructs forming all dental hard substances could be harvested. Recently, tooth buds could be generated by co-culturing postnatal dental stem cells with human HUVEC cells encapsulated in gelatine hydrogel [56]. Only postnatal dental stem cells were used by Yang et al. (2016), who differentiated odontoblasts and osteoblasts from pig dental pulp stem cells and seeded them with gingival epithelium on a bioactive scaffold. Implantation into extraction sockets of 13.5-month-old pigs revealed the development of teeth in seven of eight animals. The regenerated molar teeth expressed dentin-matrix protein-1 and osteopontin [212]. 

### 4.4. Problems in Whole Tooth Regeneration

Despite the progress in some basic strategies for tooth regeneration, we still face a lot of problems [18,48]. An important condition for a proper functional occlusion in a dentition where teeth should be replaced by regeneration is the correct anatomical size and shape of the crown. Especially the relief of the occlusal surface with its specific pattern of fissures and cusps is relevant for a functional occlusion. The proper size and shape of a crown are determined by epithelial morphogenesis forming spatially regulated cellular condensations as signaling centers, called knots [71,197]. These knots (initiation knot, primary enamel knot, and secondary enamel knot) regulate crown development and cusp number, morphology, and pattern by expressing different factors such as FGF, BMP, Wnt, or Shh, as already mentioned. The number of tooth cusps in the mouse depends on the activity of Shh, EDA, and Activin A pathways [71,197]. The tooth size is independently regulated from the cusp number and is not only dependent on epithelial, but also mesenchymal influences. Therefore, it was suggested that the tooth size could be controlled by prolonging the activity of tooth epithelial stem cells and increasing the number of mesenchymal stem cells in recombination experiments [197]. The different tooth types such as molars or incisors have specific morphological features not only of the occlusal surface but also of the crown and root morphology. This will also be an important aspect for future tooth engineering [193]. The quality and the biomechanical loading of dental hard tissues are also important for occlusion and mastication. In already developed models of tooth regeneration, only a low level of enamel mineralization could be observed. 

Tooth health is also dependent on proper vascularization and innervation. While vascularization occurs in different models already published [213], the question is whether this would be also sufficient for the long-lasting survival of regenerated teeth. Efforts have been made to induce neurogenesis and formation of nerve fibers, e.g., by using exogenous agents such as semaphorin 3 receptor inhibitors, by application of immunomodulation using cyclosporin A, or implication of bone marrow stromal cells [48]. Recently, Strub et al. recombined embryonic dental epithelium with a mixture of dental mesenchymal cells and bone marrow-derived cells and cultured and implanted these cells subcutaneously. The tooth-like tissues obtained were innervated with axons entering the newly formed pulp [214]. 

Other problems include the formation of a proper periodontium or infections occurring during or after transplantation. If whole tooth constructs can be implanted, the role of the tissue environment will play an important role in the success: How is the quality of the jawbone? How will the implantation be affected by age or systemic diseases of the patients? How resistant will the newly created tooth be against probable infections? Finally, the costs of creating a “bio-tooth” are also unpredictable yet [48,156].

## 5. Conclusions and Future Perspectives

Progress in regenerating whole teeth will need scientific research on different levels such as identification of appropriate cell sources with tooth inductive signals. For this further research on the feasibility of iPS cells for this approach is important. Furthermore, the identification of master genes in gene regulatory networks responsible for tooth induction and tooth formation is necessary for successful manipulation of, e.g., adult cells to form bioengineered dental tissues, and to control tooth crown, size and tooth identity. 

Applying the acquired knowledge about signaling pathways shaping dental tissue genesis might stimulate novel cell culture techniques establishment and functionalized scaffolds development. Functionalized biomaterials will presumably play a central role in hard dental tissue regeneration such as dentin and cementum and probably the main role in enamel regeneration since this tissue is acellular and cannot be reproduced in vitro relying solely on a cell-based approach. Although several potentially appropriate biomaterials have already been investigated and tested, only very few examples were used in clinical studies until now. Future efforts in stem cell-based approaches will very likely be directed toward biomaterials that allow sequential and on-demand drug release of multiple drugs in order to tailor timely the different cascade processes during amelogenesis, dentinogenesis, and cementogenesis, respectively. 

On the translational level, methods to improve 3D organogenesis, 3D printing applications, or the appropriate application of stimulatory molecules and drugs should be tested intensively. Solutions must be found for the proper mineralization of dental hard tissue formed by the regeneration process to ensure the natural properties of teeth in occlusion and mastication. Finally, there are considerable financial investment problems that should be taken into account. Then, but only then, whole biological tooth regeneration may even be a blueprint for the regeneration of other complex organs [70]. 

## Figures and Tables

**Table 1:** Cell Sources and signaling modulators useful for amelogenesis, dentinogenesis, and cementogenesis.

Tissue	Plausible Cell Sources	Signaling Pathway/Node	Interfering Molecule(s)	
			Stimulatory	Inhibitory
Enamel	Keratinocyte stem cells [87]; ERM from periodontal ligament [88]; OEpSCs [89]; AT-MSCs [90]; iPSCs [91,92,117]	Hh	Shh [42]; purmorphamine [118] ^a^	cyclopamine [118] ^a^
		FGF	FGF8 [87], *FGF10* [118] ^a^	pan-FGF receptor inhibitor SU5402 [118] ^a^
		Wnt/β-catenin	6-Bromoindirubin-3′-oxime (BIO) (GSK3βi) [45]	GSK3β [99], ICG-001 [97]
		BMP	BMP2/4 [21,22] ^b^	Noggin (BMP4i) [117]
		TGFβ	TGF-β1,2,3 [47,100]	*SMAD7* [119] ^a^
Dentin	DPSCs [25,81,101,102]; SHEDs [80]; AT-MSCS [102]; iPSCs [103]	Hh	Shh [23], purmorphamine [120]	_
		FGF	FGF2 [26,27,120]	PD173074 (FGFR1i) [120]
		Wnt/β-catenin	BIO, CHIR, Tideglusib (GSK3bi) [111,121] ^b^, Wnt7b [83];	XAV939 (tankyrasei) [31,101], rhDKK1 [101]
		BMP	BMP2 [28,108] *^b^*, BMP4 [108] ^b^	Noggin, LDN193189 [101]
		P2Rs	ATP, ARL 67156 (ATPasei) [32]	Suramin [32], iso-PPADS tetrasodium salt [82]
		ERK1/2	Leptin [105]	PD98059 (ERK1/2i) [105]
		ERK1/2	Amelogenin [104]	U0126 (ERK1/2i) [104]
Cementum	PDLSCs [75]; DFSCs [113]; iPSCs [114]	Wnt/β-catenin	LiCl, Wnt3a [35]	DKK1 [35]
		FGF	FGF2 [116] ^b^	_
		BMP	BMP2/4 [75]	FGF2 [75]
		TGFβ	rhTGFβ-1 [78]	SIS3 (Smad3i) [37]
		ERK1/2	Amelogenin [39], LRAP [79]	U0126 (ERK1/2i) [79]

^a^ studies of epithelial invagination/development; ^b^ studies in vivo; the rest are cell culture-based reports.

**Table 2:** Compilation of recently published studies emphasizing regenerative approaches of enamel, dentin, and cementum.

Tissue	Scaffold Material	Study Model	Results	Ref.
**Enamel**	8DSS: Oligopeptide of eight repetitive sequences of aspartate-serine-serine	In vivo model using Sprague-Dawley rats with induced caries.	Increased remineralization by 8DSS due to inhibited enamel demineralization and promoted remineralization.	[131]
	Elastin-like polypeptide functionalized with glutamic acid residues	In vitro remineralization of bovine enamel specimens by pH cycling after immersion in biomaterial solution.	Formation of a dense layer of highly orientated apatite nanorods with mechanical properties close to natural enamel and high chemical stability against acidic impacts.	[132]
	PAMAM-dendrimers with varying terminal groups: -NH2, -COOH, -OH	In vitro remineralization of bovine enamel specimens by pH cycling.	Remineralization is affected by electrostatic interactions between scaffold and enamel surface. PAMAM-NH_2_ shows the best results, followed by PAMAM-COOH.	[133]
	ACP-loaded PAMAM dendrimers functionalized with SN15 peptide sequence.	In vitro enamel remineralization by cycling immersion in artificial saliva and demineralization solution.	Evaluated biomaterial achieves 90% higher remineralization compared to control.	[134]
**Dentin**	Nanobioactive glass cements with or without Sr	In vitro evaluation of biocompatibility and differentiation of DPSCs. In vivo evaluation using an ectopic odontogenesis model and a tooth defect model in rats.	Fast release of bioactive Ca-, Sr- and Si-ions. Promotion of the odontogenic differentiation of DPSCs in vitro. More new dentin formation by Sr-containing biomaterial in vivo.	[138]
	The organic matrix of cellulose acetate, oxidized pullulan and gelatin loaded with boron-modified bioactive glass nanoparticles.	In vitro evaluation of biomineralization, biocompatibility, proliferation, and differentiation with hDPSCs.	Boron-modified bioactive glass nanoparticles exhibit promotive effects on the deposition of a CaP as well as on adhesion, migration, and differentiation of hDPSCs.	[139]
	Biphasic collagen matrix: Inner section of lower stiffness loaded with VEGF covered by an outer section of higher stiffness loaded with BMP2.	In vitro evaluation using hDPSCs regarding biocompatibility, proliferation, and differentiation.	The direction of DPSCs differentiation is regulated by material stiffness and amplified by the respective growth factor.	[140]
**Cementum**	retroMTA + tricalcium phosphate	In vivo test using dehiscence periodontal defects in dogs.	Significantly increased the new bone and cementum formation. The biodegradability of retroMTA is enhanced by adding TCP.	[143]
	Calcium phosphate loaded with BMP2	In vivo periodontitis model using critical-sized supra-alveolar defects in dogs.	Significant increase in regeneration of mineralized tissues. Loading with BMP2 leads to a further 2–3-fold increase.	[144]
	Bilayered material: FGF2-propyleneglycol alginate gel covered by BMP2-PLGA/CaP cement.	In vivo test using three wall periodontal defects in non-human primates.	Significantly enhanced regeneration of cementum and periodontal ligament. Newly formed PDL is highly vascularized.	[145]
	PCL-based bilayered material: a flexible porous membrane delivers cell sheets and is covered by a fibrous and porous 3D compartment.	In vivo test using dehiscence periodontal defects in sheep to evaluate the potential of different cell types forming the cell sheets: Gingival cells (GCs), PDLCs, and hBM-MSCs.	Scaffolds containing BM-MSCs and PDLCs show superior new bone and cementum formation compared to scaffolds containing gingival cells.	[147]

**Table 3:** Materials applicable for loading, encapsulation and drugs/signaling molecules release for promoting cell proliferation, and differentiation.

Signaling Molecule	Material for Drug Loading/Encapsulation and Release	Application	Release Efficiency/Kinetics Tested in	Reference
Amelogenin (EKR1/2 activator)	Self-assembled nanogels of cholesterol-bearing mannan as templates for hierarchical hybrid nanostructures	Amelogenin-releasing hydrogel for remineralization of enamel damage (artificial caries)	Cytotoxicity—in PDL fibroblasts; ex vivo enamel caries models of human molars	[174]
Purmorphamine (Hh activator/Smo agonist)	Glutaraldehyde (GA)-crosslinked gelatin type B matrix (for small molecules and proteins release)	In vitro delivery system for Wnt, Hh agonists and growth factors (e.g., FGF2, VEGF) beneficial for endochondral ossification	Release kinetics (burst vs. sustained release) studied without using cell culture; released molecules bioactivity verified in cell culture/biological assays	[175]
	Poly(propylene glycol–co-lactide) dimethacrylate (PPLM) adhesives for incorporating purmorphamine and TCP	Cell attachment and response to photocured, degradable bone adhesives containing TCP and purmorphamine	MC3T3-E1 (mouse pre-osteoblast cell line)	[176]
	PCL microspheres for encapsulating small molecules using a single emulsion oil-in-water method	Purmorphamine and retinoic acid-loaded microspheres for prolonged release during neural differentiation	Human iPSC aggregates differentiating into motor neurons	[177]
FGF	D-RADA16 peptide hydrogels coated on artificial bone composed of nanohydroxy-apatite/polyamide 66 (nHA/PA66) (for basic FGF release)	Porous growth factor-releasing structure for treating large bone defects	Female SD rat BM-MSCs; female SD rats with induced large bone defects	[178]
	Acetyl chitosan (chitin) gel (for binding and release of chitin binding peptide-FGF2 fusion protein)	Lysozyme-responsive (dose-dependent or activity-dependent) release of CBP-FGF2	Studies without using cell culture/biological assays	[179]
	Silk fibroin e-gel scaffolds (loaded with albumin = Fe3O4-bFGF conjugate)	Enhancing alkaline phosphatase, calcium deposition, and collagen synthesis during osteogenic differentiation	SaOS-2, osteogenic differentiation	[180]
BIO (Wnt/β-catenin activator)	Polymersomes (PMs) consisting of PEG-PCL block copolymer (approved for clinical use) loaded with BIO	BIO-loaded PMs for controlled activation of Wnt signaling and Runx2 during osteogenesis	Murine 3T3 Wnt reporter cells; Human BM-MSCs, osteogenic differentiation	[181]
	None	Local application of Wnt pathway modulators (BIO, CHIR, and Tegusib) to promote dentine regeneration	Wistar rats and CD1 mice molar damage	[121]
BMP2	Porous silica–calcium phosphate composite (SCPC50) (loaded with rhBMP2)	Sustained release of fhBMP2 for alveolar ridge augmentation in saddle-type defect	Mongrel dog with induced mandible defect	[182]
	Calcium phosphate (Ca-P)/poly(L-lactic acid) (PLLA) nanocomposites loaded with rhBMP2	3D Ca-P-PLLA scaffold sustainably releasing Ca^2+^ and rhBMP2 for enhanced osteogenesis	Human BM-MSCs, osteogenic differentiation	[183]
	Poly(lactic-co-glycolic acid)-multistage vector composite microspheres (PLGA-MSV) (for BMP2 release)	Controlled prolonged release of BMP2 for osteoinduction of rat BM-MSCs	Male SD rat BM-MSCs, osteogenic differentiation	[184]
TGF-β 1, 3	Poly(ethylene oxide terephthalate)/ poly(butylene terephthalate) (PEOT/PBT) fibrous resins for loading the growth factors	Sustained delivery of growth factors (TGF-β1, PDGF-ββ, IGF-1) using a layer by layer assembly for supporting fibroblast attachment and proliferation	TK173 (human renal fibroblast cell line), neonatal rat dermal fibroblasts (nRDFs)	[185]
	Poly(vinylidene fluoride) (PVDF) nanofibers fabricated via electro-spinning method with/without chitosan nanoparticles (loaded with TGF-β1)	PVDF-TGF-β1 as a bio-functionalscaffold for enhancing smooth muscle cells (SMC) differentiation	AT-MSCs, SMC differentiation	[186]
	Alginate nanogel with cross-junction microchannels (encapsulating TGF-β3)	Controlled release of TGF-β3 from polymeric nanogel for enhanced chondrogenesis	Human MSCs, chondrogenic differentiation	[187]
ATP, suramin (P2XR activators)	Albumin nanoparticles (aNPs) of low polydispersity loaded with ATP and coated with erythrocyte membrane (EM)	EM-aNPs developed as a delivery vehicle for ATP to be used as an anticancer agent	HeLa, HEK-293 cell lines	[188]
	Hydroxyapatite (HA)/agarose hybrids for ATP and suramin release	ATP and suramin release for hard tissue formation	Release kinetic studies without cells (see Figure 4); biocompatibility test using AT-MSCs and MG-63 cell line	[171]
